# A Rare Case of an Early Postoperative Obstructive Ileus in a Young Female Patient due to a Residual Trichobezoar Mass

**DOI:** 10.1155/2016/4121969

**Published:** 2016-04-11

**Authors:** P. Christopoulos, S. Ross-Thriepland, H. McCarthy, C. S. Day, W. Sasi

**Affiliations:** Surgical Department, Airedale General Hospital, West Yorkshire BD20 6TD, UK

## Abstract

Trichobezoar is a rare cause of small bowel obstruction, whereby a mass forms most commonly in the stomach and duodenum of young females, from ingestion of hair, a condition known as trichophagia. We present a case of recurrent small bowel obstruction due to a residual hair mass that was removed surgically in a young female patient who had a laparotomy and gastrotomy for removal of a large gastric trichobezoar just two weeks prior to the current admission. This case illustrates the importance of a thorough inspection of the whole bowel to ensure that no residual bezoars remain after surgery.

## 1. Introduction

Bezoars are concretions of indigestible matter that accumulate in the gastrointestinal tract. Trichobezoars, specifically, are defined as masses of swallowed hair usually in the stomach [1, 2] due to the smooth and indigestible nature of hair which when swallowed accumulate in the rugae of the stomach and over time, mixing with food and mucus, they become compacted and form solid masses. Most commonly, the bezoars produce obstructive symptoms but rarely may cause ulceration and even bleeding. Diagnosis is suggested by upper GI series and confirmed by endoscopy. Treatment options, as described in the literature, include enzyme therapy (papain, cellulase, and acetyl-cysteine), endoscopic fragmentation, or surgical excision.

## 2. Case Presentation

**Figure 1:**
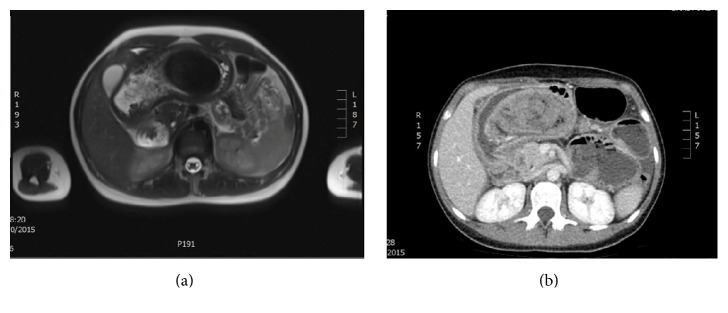
(a) MRI demonstrating two well-defined T2 hypointense and T1 isointense filling defect mass lesions within the stomach, extending into the proximal duodenum. The larger of these within the body of stomach measures approximately 100 × 50 mm (appears pedunculated) and the smaller of these in the gastric fundus measures 40 mm. (b) Preoperative CT confirms the presence of the mass in the patient's stomach.

An 18-year-old female was admitted to the surgical department with a two-week history of colicky epigastric abdominal pain, indigestion, and multiple episodes of emesis. Physical abdominal examination revealed tenderness of the epigastrium and a palpable epigastric mass which was misdiagnosed as an epigastric hernia. Patient's vital signs were normal. Rectal examination did not show any abnormal pathology. Laboratory blood tests revealed only a mild microcytic anemia and a mild leukocytosis. Multiple investigations were performed. Abdominal plain X-ray was not indicative and magnetic resonance imaging (MRI) showed two well-defined T2 hypointense and T1 isointense filling defects mass lesions within the stomach. The larger of these within the body of stomach measured approximately 100 × 50 mm and the smaller of these in the gastric fundus measured 40 mm, differentiating between gastric leiomyoma and a bezoar (Figure 1(a)). History was positive for previous trichophagia and negative for other eating disorders.

An upper GI gastroscopy test was recommended and the patient was transferred to a nearby upper gastrointestinal unit for further management where she was investigated additionally with Computed Tomography (CT) (Figure 1(b)) and had a laparotomy and removal of the gastric trichobezoar through a gastrotomy. The patient was discharged 4 days post-op.

Two weeks postoperatively the patient presented with generalized cramping abdominal and intermittent back pain, nausea, and vomiting undigested bowel content all consistent with a small bowel obstruction.

**Figure 2:**
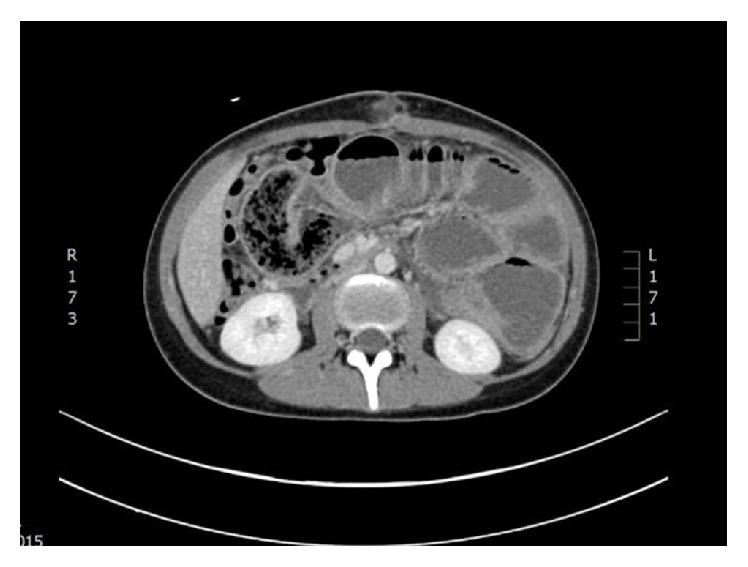
Preoperative CT showing scattered dilated loops of small bowel, measuring up to 45 mm in the left flank. Within the right upper quadrant, there is a short loop of small bowel, which contains speckled faecal matter. There is the impression of twisting in the adjacent mesentery.

On examination a distended abdomen was apparent with a palpable, hyporesonant mass in the right lower quadrant region. Abdominal X-ray was not indicative of bowel obstruction; however, CT scan imaging identified a high grade small bowel obstruction which was due to an intraluminal mass (Figure 2).

**Figure 3:**
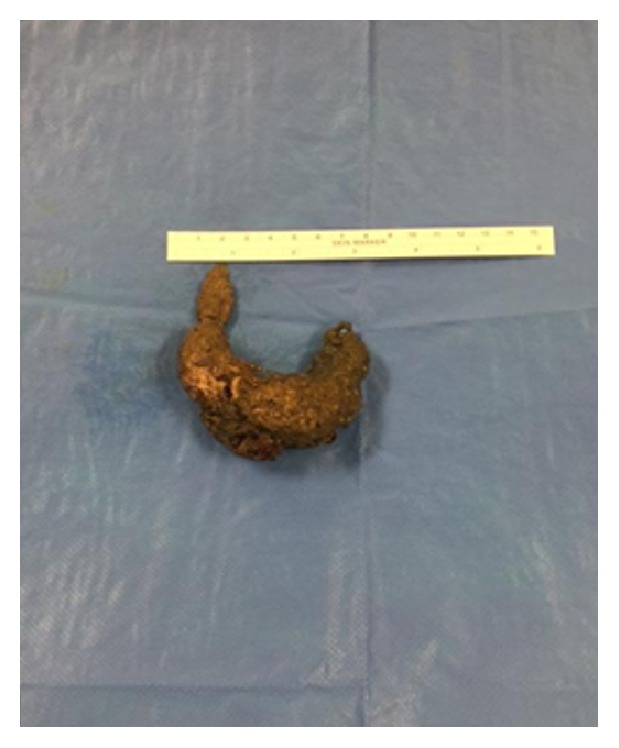
The residual trichobezoar mass recovered from the patient's small bowel.

The patient underwent an urgent laparotomy and a residual trichobezoar mass was found in the jejunum which was removed by enterotomy (Figure 3). Careful examination of the whole bowel was performed to ensure that no other satellite bezoar masses remained. The patient was discharged 5 days postoperatively after an uncomplicated recovery.

## 3. Discussion

Trichobezoar is a rare condition presenting in just 0.06–4% of the population [1] defined as a mass of swallowed hair in the stomach [1, 2]. Due to the smooth and indigestible nature of hair, when swallowed it accumulates in the gastric mucosa and over time it mixes with food and mucus, becoming compacted and forming a solid mass known as a trichobezoar [1, 3].

Presence of trichobezoar is associated with the conditions trichotillomania and trichophagia. Trichotillomania is a psychosomatic condition, involving the pulling out of one's hair. As in our case, it is most commonly seen in adolescent Caucasian females [4–6]. 5–18% of patients with trichotillomania also suffer from trichophagia from which 30% will develop a trichobezoar [7]. When the gastric bezoar extends into the duodenum and the jejunum it is called Rapunzel syndrome and it is associated with higher risk of complications [8]. Reviewing again the preoperative imaging pictures with the radiology department, the diagnosis of Rapunzel's syndrome can be excluded even though they insist on their primary report of two different masses in the stomach.

Trichophagia is often associated with mental retardation and psychiatric conditions such as personality and eating disorders [9] and these patients often have some common pathophysiological and neurobiological risk factors for another pediatric eating disorder, known as pica [9–11]. Conflicting evidence exists as to whether trichophagia and trichobezoar formation should be classified as an impulse control disorder or an affective spectrum disorder [5, 7, 10].

Although rare and usually asymptomatic [9], trichobezoar should be suspected in young females presenting with nonspecific symptoms such as epigastric pain or mass, weight loss, and fatigue. Other common signs or symptoms include vomiting, constipation, alopecia, iron deficiency, hypochromic anemia, and vitamin B12 deficiency [3, 7, 9, 12]. As bezoars increase in size they cause complications such as gastric ulceration, obstruction, perforation, pancreatitis, and obstructive jaundice [4, 12, 13]. Our patient fitted many of these symptoms which in retrospect may have been present and dismissed until she presented with an acute abdomen.

Ultrasound and abdominal radiographs are often sufficient to diagnose trichobezoar [4] but CT is the gold standard for diagnosis where trichobezoar presents as a heterogeneous mass interspersed with gas [9]. Where possible, CT is avoided in young females due to radiation risk [12] justifying our initial decision to perform an MRI test. On abdominal X-ray, trichobezoar can be identified as an opaque mass with a calcified rim or as stomach dilatation or bowel loops and air bubbles [12, 14] but in our case both times the X-rays were not diagnostic. On ultrasound it presents as hyperechoic [12]; however, multiple acoustic interfaces can make detection difficult [12, 15].

Small gastric bezoars can be removed endoscopically however; there is limited evidence of success with only a 5% success rate in a recent study [3]. Endoscopic fragmentation and enzymatic dissolution prove to be problematic due to the difficulty of fragmenting large, dense, indigestible, and solid nature of trichobezoars [1]. There is a significant risk of migration once fragmented which could result in residual satellite bezoars which can cause later obstructive phenomena. Endoscopy also poses the potential risk of iatrogenic esophageal perforation [3, 10] and is therefore used more for diagnostic reasons rather than treatment [10]. For the removal of small sized masses, laparoscopic surgery could be considered; however, it has an increased risk of spilling content into the abdominal cavity [3] and an overall success rate of just 70% in experienced laparoscopic centers [1].

According to the reviewed literature and from our recent experience, the gold standard for managing trichobezoar gastric outlet obstruction is the laparotomy, especially when it is presenting as Rapunzel's syndrome. Although highly invasive, laparotomy has the highest success rate of the methods mentioned above and is the only method whereby the whole bowel can be examined for residual and satellite bezoars and enables effective removal [3].

To the best of our knowledge there is only one other reported case of missed ileal trichobezoar that was discovered after laparotomy [1]. Even though up to 17% of cases present with multiple bezoar masses and with a rate of 80% missed at imaging it is quite surprising that there are no more similar case reports [16]. A missed bezoar mass can cause not only the complications already mentioned but also increases in the surgical complication rates by putting the patient through another laparotomy to an already scarred abdomen.

Possible explanations for residual bowel bezoar masses could be (a) endoscopic fragmentation and enzyme dissolution that could cause satellite migration of smaller hair masses [3, 10], (b) the segmentation contractions of the small bowel churning the tail of the trichobezoars in Rapunzel's syndrome [8] (this segmenting action may cause separation and migration of a smaller component with the migrating peristaltic waves of the bowel), and (c) fragmentation of the primary gastric bezoar during manipulation trying to remove it out of the stomach. In retrospect we would assume that both the second and the third mechanism could explain the residual bezoar mass.

Once more we need to highlight the importance of the exploration of the duodenum through the gastrotomy, especially if there is evidence of Rapunzel's syndrome, and the importance of a meticulous exploration of the whole bowel before closing the abdomen. In order to minimize the risk of migration of fragments while manipulating the primary hair mass occlusion of the proximal jejunum (using bowel clump) prior to gastrotomy could be a useful surgical technique.

Trichobezoar is indeed a rare cause of bowel obstruction and the diagnosis usually can be guided by a detailed history positive for coexisting psychiatric disorders. Once diagnosis is confirmed and surgical approach is necessary, it is vital that the entire gastrointestinal tract is traced via laparotomy to avoid satellite trichobezoars being missed as what happened in our case.
